# Experimental demonstration of 8190-km long-haul semiconductor-laser chaos synchronization induced by digital optical communication signal

**DOI:** 10.1038/s41377-024-01702-z

**Published:** 2025-01-08

**Authors:** Anbang Wang, Junli Wang, Lin Jiang, Longsheng Wang, Yuncai Wang, Lianshan Yan, Yuwen Qin

**Affiliations:** 1 Key Laboratory of Photonic Technology for Integrated Sensing and Communication, Ministry of Education of China, Guangdong University of Technology, Guangzhou, 510006 China; 2 Key Laboratory of Advanced Transducers and Intelligent Control System, Ministry of Education and Shanxi Province, Taiyuan, 030024 China; 3 Institute of Advanced Photonics Technology, School of Information Engineering, Guangdong University of Technology, Guangzhou, 510006 China; 4 Guangdong Provincial Key Laboratory of Information Photonics Technology, Guangdong University of Technology, Guangzhou, 510006 China; 5 College of Physics and Optoelectronics, Taiyuan University of Technology, Taiyuan, 030024 China; 6 Center for Information Photonics and Communications, Southwest Jiaotong University, Chengdu, 610031 China

**Keywords:** Nonlinear optics, Fibre optics and optical communications, Optical techniques

## Abstract

Common-signal-induced synchronization of semiconductor lasers have promising applications in physical-layer secure transmission with high speed and compatibility with the current fiber communication. Here, we propose an ultra-long-distance laser synchronization scheme by utilizing random digital optical communication signal as the common drive signal. By utilizing the long-haul optical coherent communication techniques, high-fidelity fiber transmission of the digital drive can be achieved and thus ultra-long-distance synchronization is expected. Experiments were implemented with distributed feedback lasers injected by a random-digital phase-modulated drive light. Results show that high-quality synchronization can be achieved as the drive signal rate is larger than the laser relaxation frequency and the transmission bit error ratio is below a critical value. Chaos synchronization over 8191-km fiber transmission was experimentally achieved. Compared to traditional common-signal-induced synchronization using analog drive signal such as chaos, the distance is increased by 8 times, and complicated hardware devices for channel impairment compensation are no longer required. In addition, the proposed method does not sacrifice communication capacity like traditional methods which need a channel to transmit analog drive signal. It is therefore believed that this common-digital-signal induced laser synchronization paves a way for secure backbone and submarine transmission.

## Abstract (web summary)

Long-haul chaos synchronization of semiconductor lasers commonly driven by a digital optical fiber communication signal, which enables practical physical-layer encryption.

## Graphical abstract

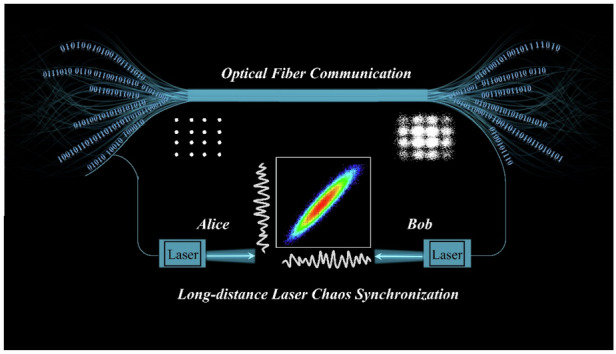

## Introduction

Physical-layer information security has been receiving extensive attention^1–6^. Optical chaos with complex waveforms which can be generated from lasers^2^, optoelectronic oscillators^7^, and microcombs^8^, enables more practical physical-layer encryption techniques including optical chaos communications^9–14^ and physical key distribution^15–23^. They have capability of compatibility with current fiber optic communication systems and potential in high encryption speed. The milestone filed experiment of optical chaos communication based on master-slave laser synchronization in Athens metropolitan area network achieved hiding transmission with 1 Gb/s data speed over a 120-km fiber link^2^. Recently, 40 Gbit/s over 800 km transmission was reported using chaotic optoelectronic oscillators with assistance of neural network^9^, and 100 Gbit/s over 100 km was demonstrated by using chaotic semiconductor lasers^10^.

Chaos synchronization^24^ that two systems generate highly similar waveforms, is the fundamental of the chaos-based encryption methods. In the last decade years, the common-signal-induced (CSI) synchronization^25–29^ of semiconductor lasers received increasing research attention due to the following advantages. First, the CSI synchronization occurs between two uncoupled response lasers which are optically injected by a common complex drive signal. This inspired a new scheme of optical chaos communication, in which the receiving laser is no longer injected by encrypted signal (i.e., the mixed chaos and message). It avoids the bandwidth limitation of chaos-pass filtering^30^ in master-slave synchronization, and thus can increase secure communication speed. Second, in the CSI synchronization the response laser outputs can be low correlated to the drive signal^25^. This feature gave birth to a new paradigm of information-theoretical secure key distribution^15–23^. Shared keys can be extracted locally from the synchronized laser outputs, and the full information of keys cannot be observed from the drive signal due to the low drive-response correlation. Various semiconductor lasers have been proven to achieve the CSI synchronization and proposed for key distribution, such as distributed feedback (DFB) lasers^25,26^, Fabry-Pérot (FP) lasers^22,27^, vertical cavity surface emitting lasers (VCSELs)^19,28^, and distributed Bragg reflector (DBR) lasers^23^. Several kinds of drive signals have also been presented, including chaotic light^19,25^, noise light^22,23^, constant-amplitude random-phase light^29^, and polarization-random light^28^. Experiments of the CSI-synchronization key distributions with key rate ranging from hundreds of kbit/s to hundreds of Mbit/s were reported over a fiber transmission longer than 100 km^16,17,22,23^.

However, the distance of laser-chaos synchronization that determines the distance of secure communication or key distribution is usually limited to a few hundred kilometers. The deterioration of synchronization quality is mainly caused by the fiber channel impairments on the analog drive signals, such as dispersion^31^, nonlinearity, and accumulated amplifier noise^32^. In 2023, the distance limit was extended to about 1000 km by optical relaying, but it requires additional hardware devices such as dispersion compensators and distributed Raman amplifiers to mitigate channel impairments^33^. It is hard to further extend the transmission distance of the analog drive signal. Moreover, the online hardware compensation also leads to changes in the fiber-link configuration, and thereby leads to challenge in compatibility with fiber communication systems. In addition, the transmission of the analog drive signal results in sacrifice of communication capacity. Therefore, the current schemes of laser chaos synchronization encounters bottleneck in long distance and complete compatibility for backbone and cross-ocean fiber secure communication.

In this paper, we propose and experimentally demonstrate an ultra-long-distance CSI chaos synchronization of semiconductor lasers by using a random digital optical communication signal as the drive. The digital drive after long-distance transmission can be recovered by using the coherent optical communication techniques, and thus high-quality long-haul chaos synchronization can be achieved with no changes to the transmission link. Furthermore, the drive itself is the communication signal and then its transmission does not reduce communication capacity like traditional methods with analog drive signals. Note that a similar idea of transmitting digital signal was used in optoelectronic oscillator synchronization^7^. There is no report on the CSI laser synchronization with digital drive signal so far, and the mechanism, conditions and how far the synchronization distance are unknown. Here, we present detailed experimental investigation and demonstration by using DFB lasers. The effects of injection parameters, rate, amplitude, and bit error rate (BER) of the digital drive signal are investigated. Results show that high-quality synchronization can be achieved with a rate larger than the laser relaxation frequency and a BER below 0.02 for a drive signal with 16-GHz baseband width. Chaos synchronization over 8191-km fiber transmission is experimentally achieved by using a 32-GBaud quadrature-amplitude modulation (QAM) signal as the drive in a coherent optical communication system. This work will open an avenue for high-speed secure transmission in backbone and submarine transmission networks.

## Results

### Principle and experimental setup

**Fig. 1:**
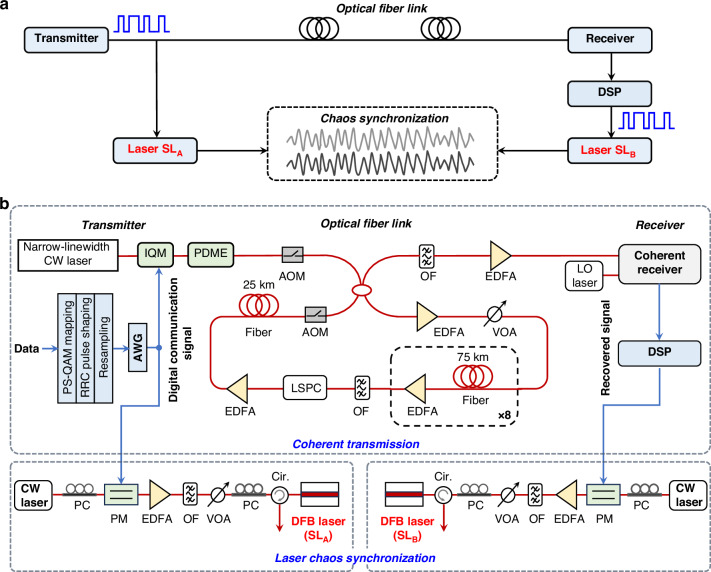
**a** Schematic diagram and **b** experimental setup of chaos synchronization of semiconductor lasers commonly driven by a digital optical communication signal. SL semiconductor laser, DSP digital signal processing, CW continuous wave, IQM in-phase and quadrature-phase modulator, PDME Polarization division multiplexing emulator, PS-QAM probabilistically-shaped quadrature amplitude modulation, RRC root raised cosine, AWG arbitrary waveform generator, AOM acousto-optic modulator, EDFA erbium-doped fiber amplifier, VOA variable optical attenuator, OF optical filter, LSPC loop synchronous polarization controller, LO local oscillator, PC polarization controller, PM phase modulator, Cir circulator

Figure 1a is the schematic diagram of chaos synchronization of semiconductor lasers commonly driven by a digital optical communication signal. The random digital signal from the communication transmitter is divided into two beams. One is injected into the semiconductor laser at the transmitter side to induce chaotic oscillation. The other beam is transmitted through a long-haul optical fiber link to the receiver side and recovered by using digital signal processing of optical communication. The recovered digital signal is then injected into the parameter-matched laser. In principle, the laser can generate chaotic light synchronous with that in the transmitter side by adjusting the optical injection conditions and the drive signal parameters. The effects of parameters on synchronization will be investigated in the next section.

The experimental setup is shown in Fig. 1b. A long-haul coherent optical communication system is used to demonstrate the laser synchronization. In the transmitter side, the optical signal is generated by the following process. A word length of 2^17^–1 pseudo-random bit sequence is mapped into probabilistically-shaped (PS) QAM with information entropy *H* = 3.5 by 2 samples/symbol. The up-sampled signals are shaped by using a square root raised cosine with a roll-off factor *β* = 0.01. Then, 32-GBaud PS-16QAM signal is generated by an arbitrary waveform generator (AWG) operating at 64 GSa/s sampling rate and 25 GHz analog bandwidth. The in-phase and quadrature-phase components are used to modulate a continuous-wave light from an external cavity laser (ECL) at ~1550 nm with ~100 kHz linewidth by an integrated LiNbO_3_ I/Q modulator. Subsequently, polarization division multiplexing emulator is applied to obtain 224 Gbit/s PDM-PS-16QAM optical signal. Then, the optical signal is launched into the circulating loop testbed with 630.146-km single-mode fibers, which consists of one 25.235 km and eight 75.6 km fiber spans under ~0.155 dB/km loss and 130 μm^2^ effective area. In the loop, amplification is provided by C-band Erbium doped fiber amplifier (EDFA) with a gain of 14 dB and a noise figure of 4.5 dB. A loop synchronous polarization controller (LSPC) is applied for the purpose of simulating polarization effects in straight-line systems. The acousto-optic modulator (AOM) is deployed as an optical switch to adjust the length of the optical fiber, enabling *N*-cycle transmission. In the receiver side, an optical filter (OF) is used to filter amplified spontaneous emission (ASE) noise. The received signal after coherent detection is sampled by a real-time digital oscilloscope with 36 GHz electrical bandwidth and 80 GSa/s sampling rate. Here, the phase-diversity homodyne receiver^34^ is adopted for coherent detection due to that it can generate the baseband signal directly without dealing with a rather high intermediate frequency like the heterodyne receiver. Finally, the transmission impairments of the digital optical signal are compensated or equalized in off-line DSP module (including chromatic dispersion and nonlinear compensation, clock recovery, polarization mode dispersion compensation, carrier frequency offset and phase recovery compensation). More details about the coherent optical communication experiments can refer to our previous work^35^. Note that, the coherent fiber transmission link used in our verification experiment is a recirculating fiber loop, which is a powerful tool to experimentally simulate a long-haul straight-line transmission link^36^. For a straight-line fiber transmission link, the system can be simplified because the AOMs and LSPC are no longer required. In addition, the DSP modules as well as OFs are required to recover digital drive signal with low BER, and then to achieve high-quality chaos synchronization.

To demonstrate chaos synchronization, the in-phase (or quadrature) components of the transmitted and recovered signals are used to modulate the phase of a CW laser light to generate highly-correlated drive optical signals. The phase modulation amplitude is calculated as *φ*_m_ = π*V*_m_/*V*_π_, where *V*_m_ is the signal amplitude applied on the modulator and *V*_π_ is the modulator’s half-wave voltage. Then, the two optical drive signals are injected into two parameter-matched single-longitudinal-mode semiconductor lasers SL_A_ and SL_B_, respectively. The injecting light polarization is matched to the laser by a polarization controller. A variable optical attenuator is inserted before the laser to adjust the injection strength, which is defined as the power ratio of injection light to the laser output. The optical frequency detuning is defined as Δ*ν*_DR0_ = *ν*_D_ − *ν*_R0_, where *ν*_R0_ is the optical frequency of the static laser without injection, and *ν*_D_ is the center frequency of the drive light.

### Laser synchronization mechanism

**Fig. 2:**
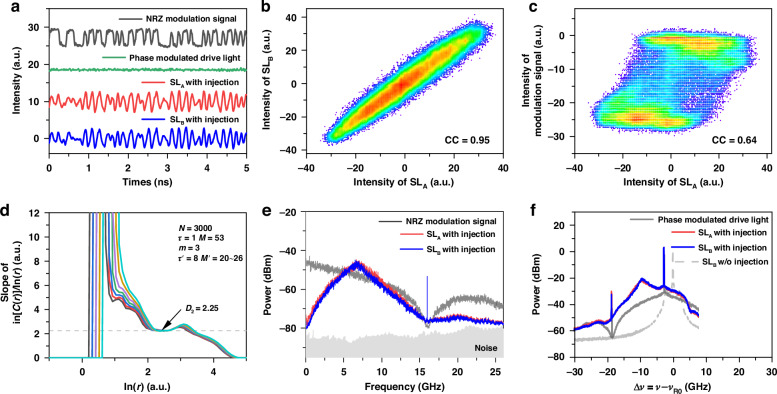
**Chaos synchronization of semiconductor lasers driven by a NRZ signal**. **a** temporal waveforms; **b** scatterplot of SL_A_ and SL_B_; **c** scatterplot of SL_A_ and NRZ modulation signal; **d** correlation dimension (*D*_2_) of SL_A_ intensity; **e** radio-frequency spectra; **f** optical spectra. *CC* correlation coefficient; the parameters in (**d**) are used for algorithm calculating *D*_2_ (see text)

We first investigate the physical mechanism and parameter conditions of chaos synchronization using non-return-to-zero (NRZ) binary codes as drive signal under back-to-back scenario. For synchronization, the two response lasers with similar threshold currents are separately biased at 14.7 mA and 14.1 mA, so that they have the same relaxation oscillation frequency of *f*_RO_ = 2.11 GHz. Their static-state wavelengths are stabilized at 1548.108 nm (see laser settings in methods). Figure 2 plots the typical experimental results which were obtained with the following parameters: injection strength *κ*_j_ = 0.4, optical frequency detuning Δ*ν*_DR0_ = –2.5 GHz, code rate *f*_m_ = 16 Gb/s, and modulation amplitude *φ*_m_ = 0.5 π. The synchronization quality is characterized by the correlation coefficient denoted as *CC* of two lasers’ intensity waveforms (see methods).

Figure 2a shows the temporal waveforms of the NRZ modulation sequence, the phase-modulated drive light, and the lasers’ response outputs. Clearly, the intensity waveforms of the lasers are almost identical, but different from that of the modulation sequence. The scatter plot of laser response waveforms is given in Fig. 2b. It concentrates around a straight line showing a high-quality synchronization, and the correlation coefficient is calculated as *CC*_AB_ = 0.95. Shown in Fig. 2c, the scatter plot of laser SL_A_ and the modulation signal shows a poor linearity with a low correlation coefficient of 0.64. These results show that the lasers can achieve synchronization under a digital drive light, and simultaneously have a low correlation to the drive signal. The low drive-response correlation is beneficial to security of synchronization-based applications such as secure communication and key distribution. Further decrease of the drive-response correlation can be implemented by introducing additional optical scrambler such as dispersion devices and microsphere cavities.

The sub-band signals of the synchronized laser waveforms are also synchronous. After applying low-pass filtering on the laser waveforms, the correlation coefficient increases quickly to 0.91 as the cutoff frequency increases to 1.5 GHz, and reach its maximum of 0.97 when the cutoff frequency is 4.8 GHz (see section S1 in the supplementary file). This means that full-bandwidth detection of laser output is not necessary for applications in key distribution. Furthermore, a low-rate key distribution is feasible with efficient detection of a low-frequency sub-band of laser chaos.

Correlation dimension analysis^37^ is implemented to demonstrate that the response laser output exhibits chaotic behaviors. To reduce the influence of detection noise, the Grassberger-Procaccia algorithm with re-embedding method^38^ is employed to estimate the correlation dimension *D*_2_ of the laser intensity waveform (See methods). Figure 2d plots the correlation integral *C*(*r*) of the laser intensity waveform obtained with embedding parameter *τ* = 1, *M* = 53, *m* = 3, *τ*′ = 8, and *M*′ = 20–26. Clearly, the curves of slope of ln[*C*(*r*)]/ln(*r*) converge to a plateau within a certain range of *r*. This means the correlation dimension is equal to the ordinate value of the plateau, that is *D*_2_ = 2.25.

Seen form Fig. 2e, the response lasers have a wide RF spectrum but dominated by laser relaxation frequency. This profile is highly similar to that of chaotic semiconductor laser with optical feedback. In addition, the spectrum of laser output is obviously different from that of the modulation code sequence, which indicates the low drive-response correlation again. Figure 2f plots the optical spectra of the response lasers and the drive light. The optical spectrum of the drive light in light gray has sidebands with 16 GHz bandwidth and side-peaks corresponding to the fundamental frequency of the NRZ modulation signal. The dashed line is the optical spectrum of the static laser. Note that the detuning of Δ*ν*_DR0_ = −2.5 GHz can be seen between the center spectral lines of the drive and the static laser. As shown in the blue and red lines, the lasers under injection have the same optical spectral profile having the following characteristics. First, the laser spectrum is broadened and wavelength red shifted. Second, the center frequency is locked to that of the drive light, but the sidebands are not locked: the negative sideband has a larger response than the positive sideband. This indicates that the mechanism of the laser synchronization is the consistent nonlinear response to the drive signal under condition of center frequency locking.

**Fig. 3:**
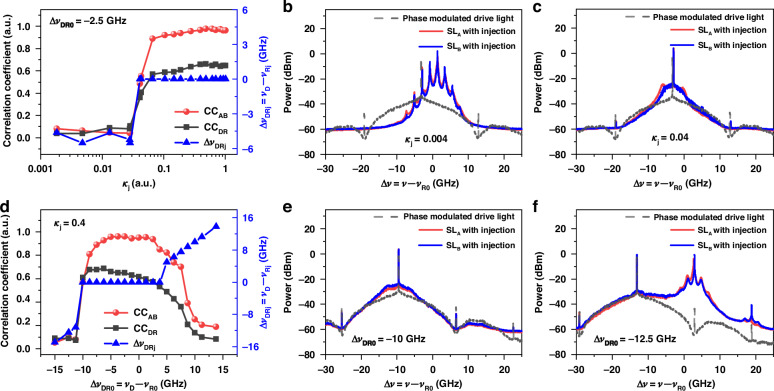
**Effects of injection parameters on chaos synchronization and frequency detuning** Δ*ν*_DRj_ = *ν*_D_ − *ν*_Rj_: **a** injection strength *κ*_j_ and **d** initial frequency detuning Δ*ν*_DR0_, where *ν*_Rj_ is the optical center frequency of the laser with injection; representative optical spectra at **b** *κ*_j_ = 0.004, Δ*ν*_DR0_ = −2.5 GHz, **c** *κ*_j_ = 0.04, Δ*ν*_DR0_ = −2.5 GHz, **e** Δ*ν*_DR0_ = −10 GHz, *κ*_j_ = 0.4; **f** Δ*ν*_DR0_ = −12.5 GHz, *κ*_j_ = 0.4

To further reveal synchronization mechanism and conditions, effects of injection parameters are investigated. We measure another frequency detuning Δ*ν*_DRj_ = *ν*_D_ − *ν*_Rj_ to indicate whether center frequency locking happens, where *ν*_Rj_ is the center frequency of the laser with optical injection. Figure 3a shows the effects of injection strength *κ*_j_ on Δ*ν*_DRj_, *CC*_AB,_ and *CC*_DR_ in triangles, circles, and squares, respectively, with an initial detuning Δ*ν*_DR0_ = −2.5 GHz. As *κ*_j_ < 0.03, Δ*ν*_DRj_ fluctuates around −5 GHz meaning that lasers are unlocked, and both *CC*_AB_ and *CC*_DR_ have a small fluctuation around 0.1. Representative optical spectra of lasers under weak injection of *κ*_j_ = 0.004 are given in Fig. 3b. The laser spectra have multiple peaks which are induced by frequency mixing effect of injection light and laser mode. In addition, the laser spectra have no gain in the left sideband of drive light. The above two features indicate that the lasers have little response to the modulated NRZ signal and their dynamics are mainly induced by the CW light carrier. This is the reason of that the laser response has no correlation to the drive signal. The low correlation between two lasers is mainly caused by their inner spontaneous emission noises which are naturally different.

As *κ*_j_ increases to 0.04, the lasers begin to exhibit locking phenomenon, i.e., Δ*ν*_DRj_ = 0 GHz. Shown in Fig. 3c, the center frequency of lasers is locked to the drive light, and the laser spectra have no other peaks like Fig. 3b. In this case, *CC*_DR_ and *CC*_AB_ approximately increase to 0.3 and 0.5, respectively. This means that the lasers have response to the modulated digital drive signal but are still not synchronized. The reason is that effect of inner spontaneous emission noise on the laser dynamics is not negligible compared to the weak drive signal, and then causes dissimilarity between two lasers’ response. Further increasing injection strength of the common drive signal can suppress the noise-induced dissimilarity and thus achieve laser synchronization. Seen form Fig. 3a, chaos synchronization with *CC*_AB_ > 0.90 can be achieved as *κ*_j_ > 0.06.

Figure 3d displays the effects of initial frequency detuning Δ*ν*_DR0_ on Δ*ν*_DRj_, *CC*_AB,_ and *CC*_DR_ under *κ*_j_ = 0.4. The injection locking occurs as the initial detuning Δ*ν*_DR0_ ranges from −10 GHz to 3.75 GHz, while the laser synchronization with *CC*_AB_ > 0.90 is observed in a range of −6.25 GHz to 2.50 GHz. It is found again that the injection locking region of Δ*ν*_DR0_ is wider than the chaos synchronization region. As shown in Fig. 3e, the lasers’ optical spectra at Δ*ν*_DR0_ = −10 GHz and *κ*_j_ = 0.4 are like that in Fig. 3c obtained under weak injection *κ*_j_ = 0.04 and small detuning Δ*ν*_DR0_ = −2.5 GHz. The laser is locked in center frequency but has weak response to the modulation sideband of drive light. Shown in Fig. 3f, for a larger initial detuning such as −12.5 GHz, the lasers are no longer locked to the center frequency of the drive light. They have a strong response to the side peak near to the laser’s static frequency, which corresponds to the modulation frequency of digital signal. Therefore, one can find that large initial frequency detuning will make the modulation sideband of drive light appear at or outside the edge of the laser gain spectrum, and thus cannot induce laser synchronization. It is equivalent to reducing injection strength. Together, these results reveal that chaos synchronization occurs under the condition of center frequency locking and strong nonlinear response to modulation sideband of the drive light.

### Effects of digital drive signal on laser chaos synchronization

**Fig. 4:**
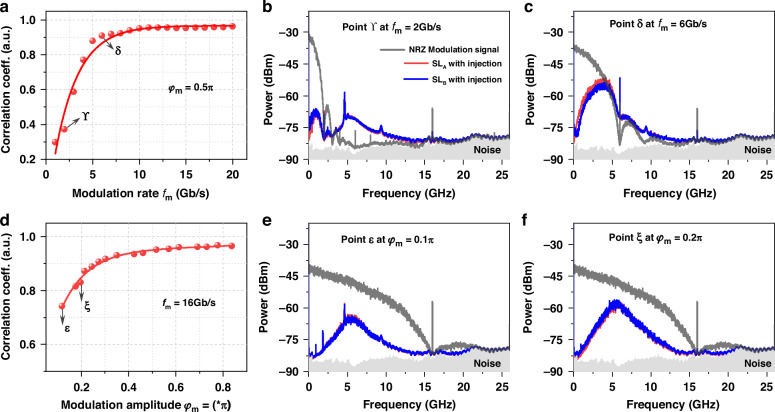
**Effects of modulation parameters on synchronization coefficient**. **a** modulation rate *f*_m_ and **d** modulation amplitude *φ*_m_. Representative RF spectra at **b** *f*_m_ = 2 Gb/s, *φ*_m_ = 0.5π, **c** *f*_m_ = 6 Gb/s, *φ*_m_ = 0.5π, **e** *f*_m_ = 16 Gb/s, *φ*_m_ = 0.1π; **f** *f*_m_ = 16 Gb/s, *φ*_m_ = 0.2π

Figure 4a plots the influence of modulation rate *f*_m_ of digital signal on synchronization quality, obtained with *φ*_m_ = 0.5π. It is found that *CC*_AB_ increases rapidly as the modulation rate increases, and exceeds 0.90 as *f*_m_ > 4.2 GHz. Synchronization coefficient *CC*_AB_ saturates at about 0.95 as *f*_m_ > 10 Gb/s. The tendency can be understood as follows. For *f*_m_ = 2 Gb/s, shown in Fig. 4b, the lasers’ RF spectra have a sub-band within 2 GHz. The sub-band components are the laser response to the drive signal and are thus synchronized. However, the laser spectra also have a higher and narrower peak at about 4.2 GHz, which is the relaxation oscillation excited by the phase jump (acting as an impulse) of the drive light. Affected by laser intrinsic noise, the excited relaxation oscillations of two lasers are not synchronized. As a result, the two lasers are low correlated with a correlation coefficient of 0.52. With increase of *f*_m_, the sub-band become wider and stronger, and thus the correlation coefficient between lasers rises. When *f*_m_ is larger than the relaxation frequency, the components of relaxation oscillation of two lasers are forced to be synchronized by the drive signal, and then high-quality chaos synchronization is achieved. Shown in Fig. 4c obtained with *f*_m_ = 6 Gb/s, the laser response spectra are identical and have no narrow peak at relaxation frequency. The corresponding correlation coefficient is about 0.90. Note that, the laser relaxation frequency after optical injection is larger than that of the solitary laser.

Figure 4d depicts the effects of modulation amplitude on synchronization quality, obtained with *f*_m_ = 16 Gb/s. One can find that *CC*_AB_ increases as the modulation amplitude rises and exceeds 0.90 as *φ*_m_ > 0.27π. For a small modulation amplitude, the injection of drive light is approximate to continuous-wave injection. For instance, shown in Fig. 4e, the RF spectrum of laser response under *φ*_m_ = 0.1π has a narrow peak at relaxation frequency. This peak will disappear with increase of modulation amplitude, for example *φ*_m_ = 0.2π shown in Fig. 4f. Therefore, large-amplitude common drive signal can induce more complex laser dynamics and simultaneously can suppress the noise-induced dissimilarity to realize high-quality synchronization.

Like synchronization quality, chaos bandwidth is also affected by injection strength and modulation rate of drive signal (see section S2 in the supplementary file). In our experiments, the chaos bandwidth still can rise monotonically from about 4 GHz to 9 GHz in the chaos synchronization region with increase of the injection strength or the modulation rate of drive signal. These results indicate that bandwidth-tunable chaos synchronization is obtained. Note that, the chaos bandwidth cannot be further increased due to the limit of the relaxation oscillation frequency.

**Fig. 5:**
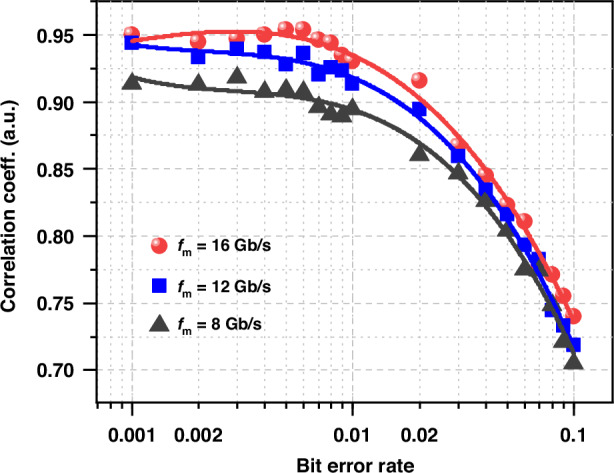
**Influence of bit error rate of modulation signal at different modulation rates**. *κ*_j_ = 0.4, Δ*ν*_DR0_ = −2.5 GHz, *φ*_m_ = 0.5π

Error bits will be inevitably introduced after transmission due to fiber channel impairments. In the back-to-back experiment, we added error bits to the drive signal for one response laser to investigate effects of BER. Figure 5 shows the effects of BER of the drive signals on chaos synchronization at different digital signal rates *f*_m_ = 16, 12, and 8 Gb/s. The results of all the three cases agree that *CC*_AB_ initially remains at a high level, but then decreases rapidly once BER exceeds a critical value. For example, the critical BER values to achieve *CC*_AB_ = 0.9 are 0.007 and 0.015 for *f*_m_ = 8 Gb/s and 12 Gb/s, respectively. If the BER of an optical communication system is lower than the critical value required for synchronization, the communication signal can be used as drive to realize laser synchronization. In addition, the critical BER increases as the increase of drive signal speed. Note that, for *f*_m_ = 16 Gb/s the critical BER is slightly larger than 0.02, which is the soft threshold criterion of the Forward Error Correction. This means that high speed optical communication system with a symbol rate not smaller than 16 GHz can be suggested for long-distance common-digital-signal induced laser chaos synchronization.

### Experimental result of 8191-km laser chaos synchronization

**Fig. 6:**
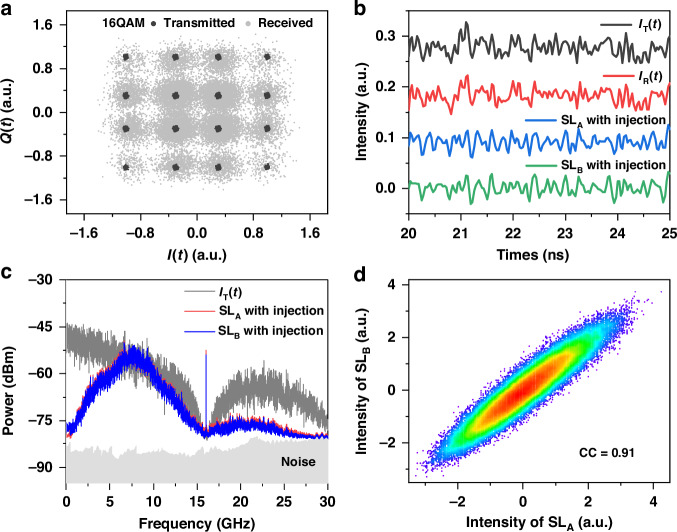
**Experimental results of chaos synchronization over 8191-km fiber link**. **a** Constellation diagram of PS-16QAM signal before and after transmission; **b** temporal waveforms; **c** radio-frequency spectra; **d** scatterplot between intensity waveforms of SL_A_ and SL_B_

Finally, the coherent optical communication system depicted in Fig. 1b is used to verify the long-distance chaos synchronization experimentally. The transmitted is a 32-GBaud PS-16QAM optical signal. The length of the circulating fiber loop in the fiber link was 630.146-km. As the optical signal propagates 13 cycles in the fiber loop, the total fiber transmission length is ~8191-km, and the corresponding BER is 0.014 which satisfies the synchronization condition. Figure 6a shows the constellation diagram of the PS-16QAM signal before and after fiber transmission represented by black and gray dots, respectively. Obviously, after fiber transmission the constellation points exhibits a decentralized and overlapped distribution. The temporal waveforms of the in-phase components I_T_(*t*) and I_R_(*t*) of the transmitted and received QAM signals are plotted in the upper part of Fig. 6b. Clearly, they have a high similarity. Two drive lights were remotely generated by using I_T_(*t*) and I_R_(*t*) to modulate the phase of CW light, and then separately injected into the two response lasers. As shown in the lower part of Fig. 6b, the intensity waveforms of the two lasers SL_A_ and SL_B_ exhibit high similarity. Figure 6c displays the radio-frequency spectra of the laser response outputs as well as that of the in-phase component I_T_(*t*) of the 32-GBuad PS-16QAM signal. Note that the baseband width of the 32-GBuad QAM signal is narrowed to about 16.16 GHz due to the square root raised cosine. The spectral profiles of the lasers are highly consistent with each other but they are noticeably different from that of I_T_(*t*). Displayed in Fig. 6d, the scatterplot of the intensity waveforms of SL_A_ and SL_B_ shows a linear correlation, and the correlation is calculated as *CC*_AB_ = 0.91. This proves that the fiber transmission distance for high-quality laser chaos synchronization can reach 8191-km. The synchronization distance can be further increased by using optical communication signal with a higher Baud rate or a wider baseband. It is worth noticing that a recent experiment^39^ realized coherent optical communication over 16,500 km fiber transmission with a BER of 0.02. It is therefore believed that the laser synchronization distance can be further extended to the same level.

The above results are obtained by using 16QAM drive signals with a BER of 0.014 and a baseband width of 16.16 GHz. By comparison, the synchronization coefficient for 16-Gb/s NRZ drive signals with a BER of 0.014 is estimated from Fig. 5 as about 0.92. It is therefore deduced that NRZ and advanced-modulation-format drive signals can obtain similar synchronization coefficient, provided they have the same baseband width and BER.

It is worth noting that advanced-modulation-format signals are beneficial to reducing the probability of transient synchronization interruption. The interruption is caused by the special case that multiple identical modulation levels occur consecutively, such as 0000 or 1111, and the corresponding duration time is larger than the laser relaxation period (see Section S4 in Supplementary Information). In this case, the drive light is equivalent to a CW light without phase modulation, and thus the lasers tend to a stable state so that chaos synchronization is interrupted. For the same baseband width, QAM signals have more and shorter modulation levels than NRZ format, and thus can effectively reduce the probability of this synchronization interruption. We also note that QAM is more susceptible to noise. According to refs. ^40,41^, to satisfy the BER condition of chaos synchronization, the required optical signal-to-noise ratio (OSNR) in theory will be increased about 6–7 dB as modulation format changes from QPSK to 16QAM and from 16QAM to 64QAM. Therefore, usage of high order QAM signal as drive leads to a high requirement of OSNR or a shortened transmission distance.

## Discussion

Although experimentally demonstrated for DFB lasers, the long-distance chaos synchronization induced by common digital communication signal is also suitable for other semiconductor lasers such as DBR lasers and VCSELs. Note that, the laser relaxation frequency determines not only the required baseband of drive signal but also the laser chaos bandwidth; the chaos bandwidth determines the physical key generation speed^15,22,23^. Usually, the relaxation frequency of DBR lasers that have longer active length is usually smaller than that of DFB and VCSEL lasers^42^. Shortening the active cavity length is an effective way to increase relaxation frequency, and for example, a relaxation frequency of 10 GHz for DBR lasers^43^, about 14 GHz for VCSEL^44^ or DFB lasers^45^ were reported. A kind of distributed reflector (DR) lasers^46^ consisting of a short-cavity DFB laser and a DBR reflector has a higher modulation speed due to photon-photon resonance effect. The nonlinear dynamics and chaos synchronization of the DR lasers is worthy for further study in future.

Chaos synchronization is affected by mismatch of inner and external operational parameters of two lasers. Our experiments show that the mismatch tolerance of laser center frequency is about from −3 GHz to 4.75 GHz for synchronization coefficients beyond 0.9 (see Section S3 in Supplementary Information). By comparison, the chaos synchronization is more sensitive to laser intrinsic parameters than external operational parameters. The tolerable mismatch is about −0.20%–0.52% for transparency carrier density, −1.81%–3.52% for linewidth enhancement factor, and −1.38%–0.85% for linear gain coefficient, respectively (see Section S3 in Supplementary Information). The results are similar to that of FP laser synchronization induced by a common ASE noise light^22^. The strong sensitivity to intrinsic parameters means high security because it increases the difficulty for eavesdroppers to obtain a well-matched laser. The relatively-low sensitivity to operational parameters is beneficial for the robustness of synchronization.

In additon, the response laser linewidth will affect synchronization quality. In priciple, the larger laser linewidth means stronger spontaneous noise, and then leads to increase of dissimilarity or decrease of synchronization quality. Fortunately, demonstrated in our experiments, the use of response lasers with a linewidth of 1 MHz can realize high-quality chaos synchronization. This requirement of laser linewidth can be readily satisfied by using commercial semiconductor lasers.

In conclusion, we propose and experimentally demonstrate a scheme of ultra-long-distance chaos synchronization of semiconductor lasers commonly driven by a random digital optical communication signal. The mechanism of the common-digital-signal induced laser synchronization is revealed as the lasers’ consistent nonlinear response to the drive signal under the condition of center frequency locking. Requirements of rate, amplitude, and transmission BER of the digital drive signal are explored for yielding high-quality chaos synchronization. By using 32-GBaud PS-16QAM signal in an optical coherent communication system as the drive signal, ultra-long-distance laser chaos synchronization over 8191-km fiber is experimentally achieved with a synchronization coefficient of 0.91 under a transmission BER of 0.014. Compared to the CSI laser synchronization using analogy chaotic drive signal, the distance is increased by 8 times, and complicated devices for channel impairment compensation are no longer needed. The synchronization distance can be further extended beyond 16,000 km along with the improvement of coherent optical communication distance^39^. Besides, in the proposed method the transmission of digital drive signal does not reduce optical communication system capacity like traditional methods. This common-digital-signal induced laser synchronization can open a new avenue for ultra-long-distance chaos secure communication and key distribution for secure backbone and submarine transmission.

## Materials and methods

### Cross-Correlation coefficient calculation

The correlation coefficient *CC* is the maximum value of cross-correlation function

1 $$CC=\,\max \left\{\frac{ < [x(t)- < \,x(t) > ][y(t-{\tau }_{{\rm{d}}})- <\, y(t-{\tau }_{{\rm{d}}}) > ] > }{\sqrt{ < {[x(t)- < \,x(t) > ]}^{2} > < {[y(t-{\tau }_{{\rm{d}}})- <\, y(t-{\tau }_{{\rm{d}}}) > ]}^{2} > }}\right\}$$

where *x*(*t*) and *y*(*t*) represent temporal waveforms, variable *τ*_d_ denotes the delay time, and <·> denotes time averaging with a data length of 1000 ns.

### Correlation dimension analysis

We briefly introduce the Grassberger-Procaccia algorithm with re-embedding method^38^ as follows. A recorded time series with *N* data points is converted to a series of *M*-dimensional vectors by using time-delay embedding with delay *τ* and embedding dimension *M*. Then, the first *m* principal components of the *M*-dimensional vector series are calculated and used to reconstruct phase space by time-delay embedding again with re-embedding dimension *M*′ and delay *τ*′. Note that only principal components significantly higher than noise level are used for phase space re-embedding. Last, calculate the correlation integral *C*(*r*) on the re-embedding phase space, which is defined as a normalized count of the number of close pairs of points with distance smaller than *r* in the space. If the slope of ln[*C*(*r*)]/ln(*r*) converges to a positive value for a certain range of *r*, the correlation dimension *D*_2_ exists and is equal to this value. In our calculations, the correlation integral results of the laser intensity waveform were obtained with *τ* = 1, *M* = 53, *m* = 3, *τ*′ = 8, and *M*′ = 20~26.

### Settings and measurements of response lasers

In experiments, the lasers SL_A_ and SL_B_ are discrete-mode lasers (Eblana EP1550-0-DM-B05) with threshold currents of 12.8 mA and 12.1 mA, respectively. They are separately biased at 14.7 mA and 14.1 mA by current sources (ILX Lightwave LDX-3412) with a precision of 0.1 mA. The lasers’ linewidths are 1 MHz and their static-state wavelengths are stabilized at 1548.108 nm by temperature controllers (ILX Lightwave LDT-5412) with a precision of 0.1 °C. The digital drive signal is generated by an AWG (Keysight M8195A) with a sampling rate of 64 GSa/s and a bandwidth of 25 GHz. The CW laser (Yenista Tunics T100S-HP) used for phase modulation has a linewidth of 400 kHz. The phase modulators (Eospace PM-0S5-10) have a 3 dB bandwidth of 10 GHz and a half-wave voltage *V*_π_ = 3.8 V. The outputs of response lasers are measured by an optical spectrum analyzer (APEX AP2060A), an electric spectrum analyzer (R&S FSW-50) and a real-time oscilloscope (Lecroy Labmaster10ZI) with 50-GHz-bandwidth photodetectors (Finisar XPDV2120RA).

## Supplementary information

Supplementary Information for Experimental demonstration of 8190-km long-haul semiconductor-laser chaos synchronization induced by digital optical communication signal

## Data Availability

The data that support the plots within this paper and other finding in this study are available from the corresponding author upon reasonable request.
