# Supplementary material for: Experimental demonstration of 8190-km long-haul semiconductor-laser chaos synchronization induced by digital optical communication signal
Source: Light Sci Appl. 2025 Jan 8;14:40. doi: 10.1038/s41377-024-01702-z (PMC11707252; doi:10.1038/s41377-024-01702-z)
Supplement: Supplementary file 1 — Supplementary Information for Experimental demonstration of 8190-km long-haul semiconductor-laser chaos synchronization induced by digital optical communication signal [file 41377_2024_1702_MOESM1_ESM.doc]

**Supplementary Information for**

**Experimental demonstration of 8190-km long-haul semiconductor-laser chaos synchronization induced by digital optical communication signal**

Anbang Wang^1,2,3,4^, Junli Wang ^2,5^, Lin Jiang^6^, Longsheng Wang ^2,5^, Yuncai Wang^1,3,4*^, Lianshan Yan^6*^, Yuwen Qin^1,3,4^

^1^ Key Laboratory of Photonic Technology for Integrated Sensing and Communication, Ministry of Education of China, Guangdong University of Technology, Guangzhou 510006, China

^2^ Key Laboratory of Advanced Transducers and Intelligent Control System, Ministry of Education and Shanxi Province, Taiyuan 030024, China;

^3^ Institute of Advanced Photonics Technology, School of Information Engineering, Guangdong University of Technology, Guangzhou 510006, China;

^4^ Guangdong Provincial Key Laboratory of Information Photonics Technology, Guangdong University of Technology, Guangzhou 510006, China;

^5^ College of Physics and Optoelectronics, Taiyuan University of Technology, Taiyuan 030024, China;

^6^ Center for Information Photonics and Communications, Southwest Jiaotong University, Chengdu 610031, China

*Corresponding author: Y.C. Wang, E-mail: [wangyc@gdut.edu.cn](mailto:wangyc@gdut.edu.cn); L.S. Yan, E-mail: [lsyan@swjtu.edu.cn](mailto:lsyan@swjtu.edu.cn)

**S1 Analysis of correlation coefficient for different frequency components of two response lasers**

The correlation coefficient of different frequency bands between two lasers is shown in Fig. S1. These results are obtained by applying Butterworth bandpass filter (BPF) and low-pass filter (LPF) on the response laser intensity waveforms in Fig. 2. Shown in Fig. S1a, as the center frequency of BPF (with a bandwidth of 0.25 GHz) ranges about from 1.3 GHz to 12.2 GHz, the sub-band components of two lasers can reach a synchronization coefficient beyond 0.9. The maximum correlation coefficient appears at laser relaxation oscillation frequency. In contrast, the correlation coefficients for high-frequency components are markedly small. This is because high-frequency components of the laser response have a lower energy, so that high-frequency noise leads to obvious dissimilarity. Similarly, the correlation coefficient of the low-frequency components is also slightly reduced. For low-pass filtering, as shown in Fig. S1b, the synchronization coefficient beyond 0.91 is achieved as the LPF cutoff frequency increases to 1.5 GHz. When the cutoff frequency is about 4.8 GHz, the synchronization coefficient reaches its maximum of 0.97.


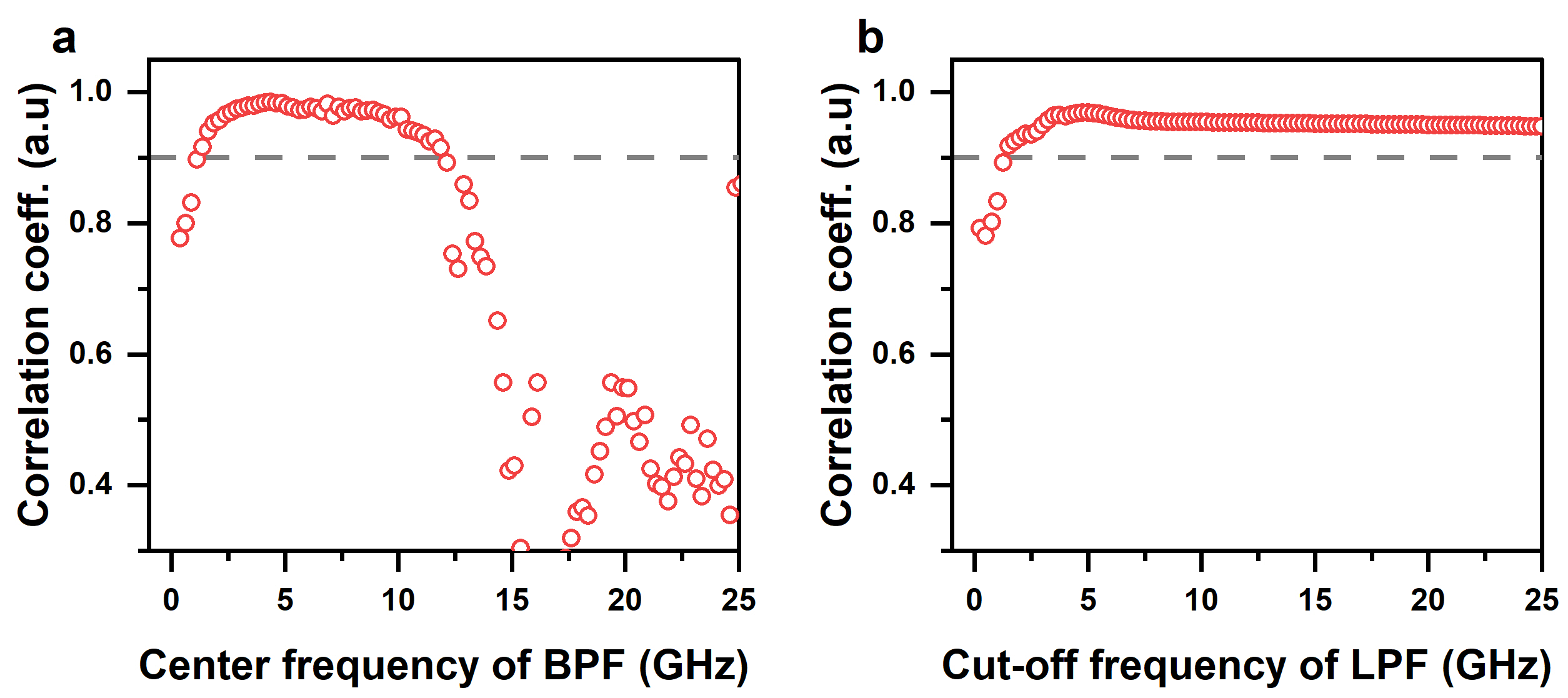


Fig. S1 Correlation coefficient under **a** bandpass filtering (BPF) and **b** low-pass filtering (LPF). *κ*_j_=0.4, Δ*ν*_DR_=−2.5 GHz, *f*_m_=16 Gb/s, *φ*_m_=0.5π.

**S2 Impact of injection intensity and modulation rate on chaos bandwidth of the response laser**

The impact of injection intensity and modulation rate on chaos bandwidth are presented in Fig. S2a and Fig. S2b, respectively. The experimental conditions of Fig. S2a and Fig.S2b are the same as that of Fig. 3a and Fig. 4a, respectively. The chaos bandwidth is evaluated as the RF spectrum span between the DC and a frequency where 80% energy is contained^1^.

Seen from Fig. 3a, the correlation coefficient quickly jumps from a low plateau to a high one as the injection strength increases, meaning chaos synchronization is achieved as *κ*_j_>0.06. It is found from Fig. S2a that, within the region of synchronization, the chaos bandwidth can rise monotonically from about 5 GHz to 9 GHz with increase of the injection strength. This can be understood as follows. The larger the injection strength is, the stronger the laser’s nonlinear response is. Thus, once the laser response becomes stronger enough to suppress the noise-induced dissimilarity, a high-quality chaos synchronization occurs and the correlation coefficient jumps upon; but the chaos bandwidth still increases gradually. Similarly, with increase of modulation rate, the chaos bandwidth also grows monotonically, as shown in Fig. S2b. These results mean that bandwidth-tunable chaos synchronization is obtained.


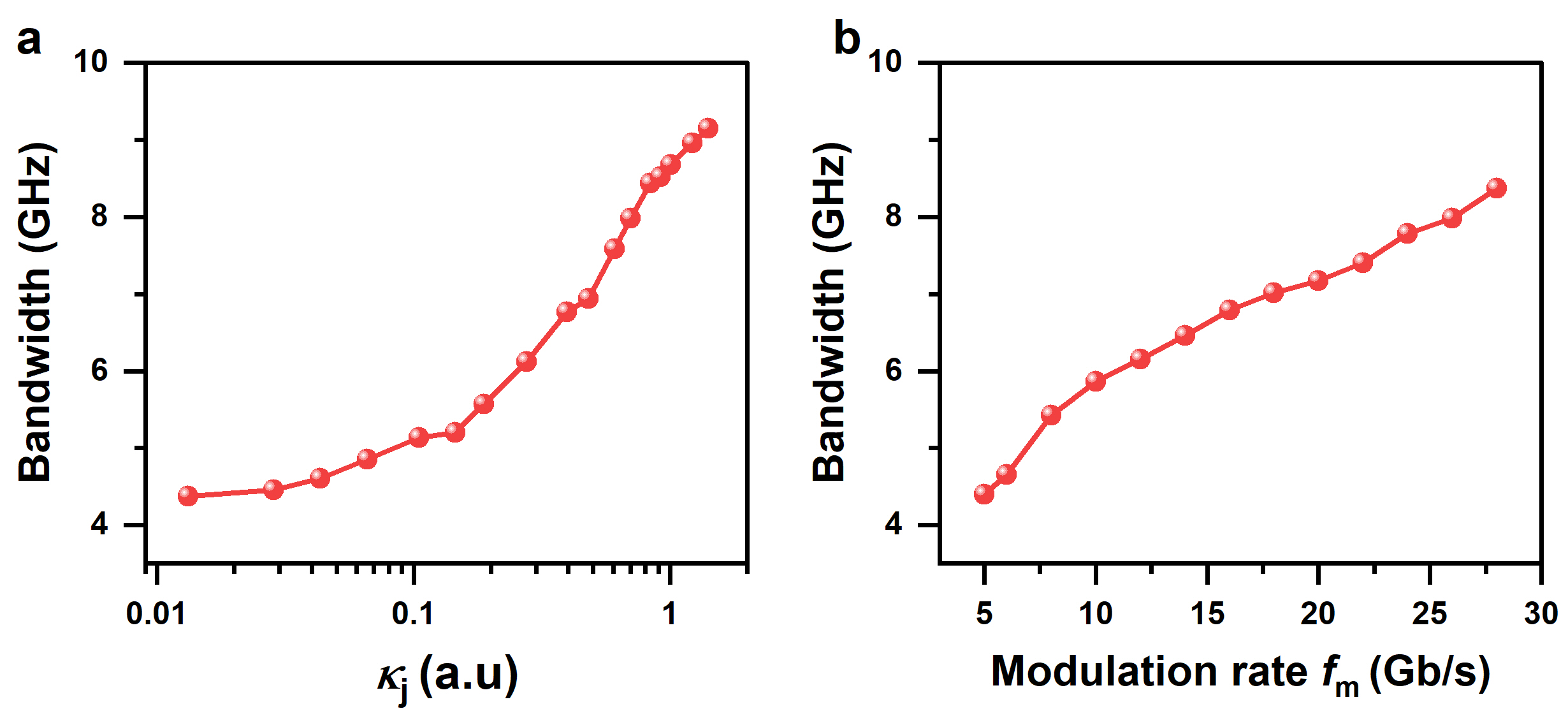


Fig. S2 Effects on chaos bandwidth: **a** injection intensity *κ*_j_, **b** modulation rate *f*_m_. Δ*ν*_DR_=−2.5 GHz, *φ*_m_=0.5π.

**S3 Effects of laser parameter mismatches on chaos synchronization**

Chaos synchronization is affected by mismatch of inner and external operational parameters of two lasers. The effects of mismatch in operational parameters (injection strength and center frequency) on synchronization coefficients is experimentally measured, as shown in Fig. S3a-b. These results are obtained by adjusting the parameters of laser SL_B_ while fixing parameters of laser SL_A_, under injection strength *κ*_j_=0.4, modulation rate *f*_m_=16 Gb/s and modulation amplitude *φ*_m_=0.5π. One can find that when the injection strength mismatch within the range of −22.71% to 48.06%, or the center frequency mismatch from −3 GHz to 4.75 GHz, the correlation value exceeds 0.90.


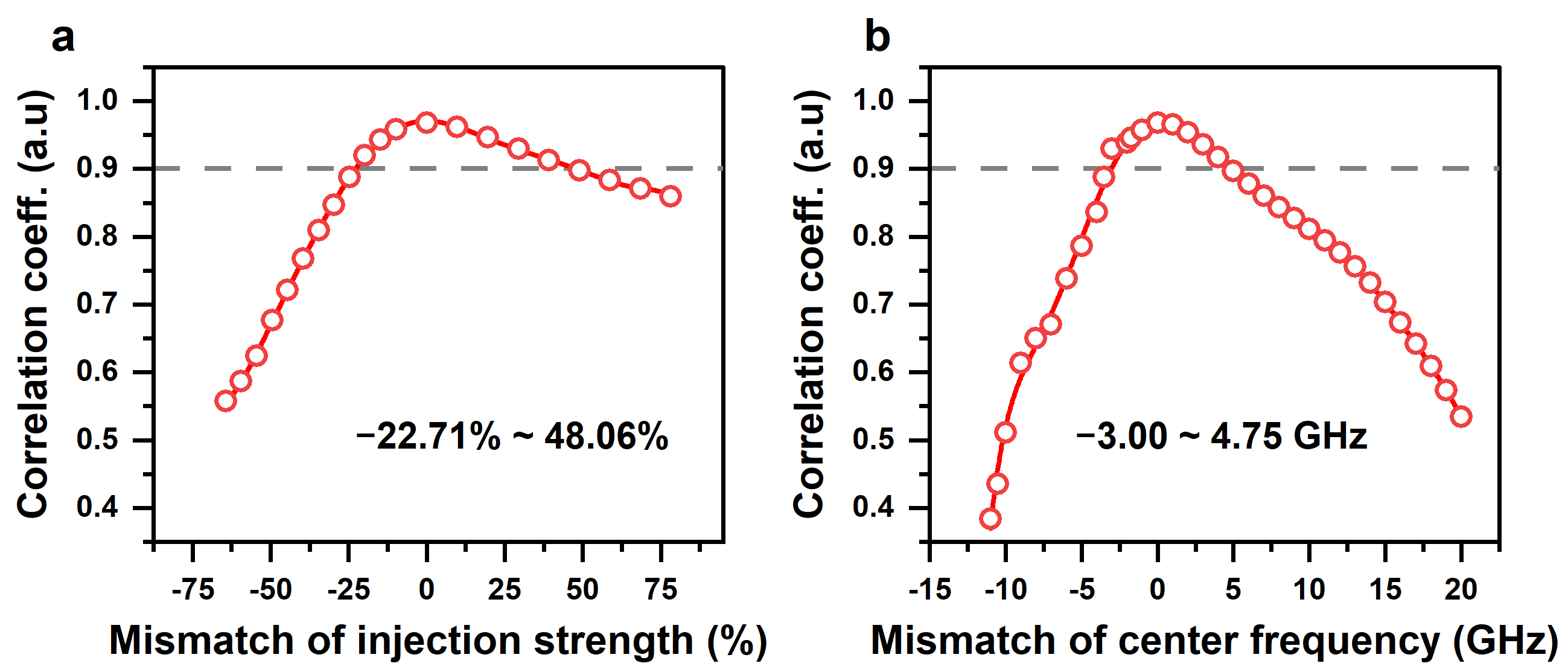


Fig. S3 Effects of external parameters mismatch on chaos synchronization: **a** injection strength mismatch, **b** center frequency mismatch. *f*_m_=16 Gbits^-1^, *φ*_m_=0.5π.

The sensitivity of chaos synchronization to laser inner parameters mismatch is analyzed by numerical simulation. The simulation system was constructed using the photonic transmission-line modelling method in VPIphotonics software^2^. The internal parameter values of lasers are listed in Table S1, and the modulation rate *f*_m_=16 Gb/s, modulation amplitude *φ*_m_=0.5π. As shown in Fig. S4a-c, the correlation value reaches a maximum of 0.96 when all the parameters are matched. The tolerable mismatches for synchronization coefficients beyond 0.9 are −0.20%~0.52% for transparency carrier density *N*_0_, −1.81%~3.52% for linewidth enhancement factor *α*, and −1.38%~0.85% for linear gain coefficient *G*_g_, respectively. By comparison, the chaos synchronization is more sensitive to laser intrinsic parameters than external operational parameters. The strong sensitivity to intrinsic parameters means high security because it increases the difficulty for eavesdroppers to obtain a well-matched laser. Only a few matched-pairs out of dozens of lasers prove to be matched with a parameter mismatch below 5% even from the same fabrication wafer^3^. The relatively-low sensitivity to operational parameters is beneficial for the robustness of synchronization.

Table S1 Simulation parameters of chaos synchronization

| Symbol | Parameter | Value |
| --- | --- | --- |
| *N*_0_ | Transparent carrier density | 1.5×10^24^ m^-3^ |
| *α* | Linewidth enhancement factor | 3.0 |
| *ε* | Gain saturation coefficient | 1×10^-23^ m^3^ |
| *Τ*_g_ | Grating period | 200.0×10^-9^ m |
| *G*_g_ | Linear gain coefficient | 3×10^-20^ m^2^ |
| *L* | Length of active region | 350 μm |
| *W* | Width of active region | 2.5 μm |
| *B*_n_ | Spontaneous emission noise bandwidth | 10 THz |
| *β* | Spontaneous emission noise coefficient | 1×10^-4^ |
| *n* | Group refraction index | 3.7 |
| *I* | Bias current | 1.5*I*_th_ |
| *f*_RO_ | Relaxation oscillation frequency | 2.32 GHz |


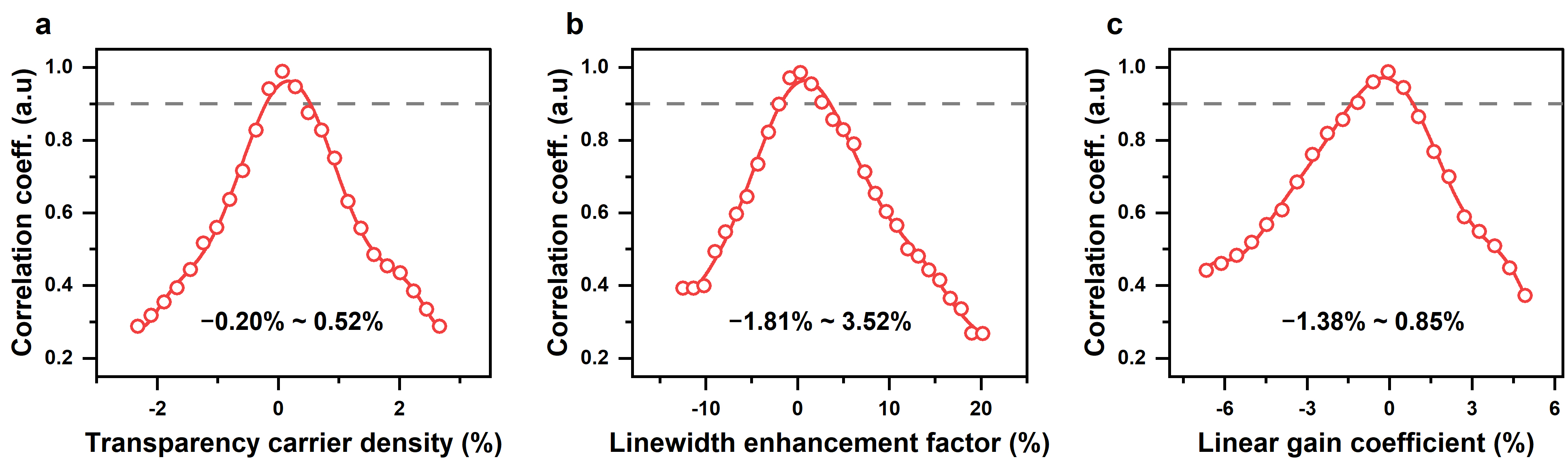


Fig. S4 Influence of internal parameter mismatch on synchronization: **a** transparent carrier concentration *N*_0_; **b** linewidth enhancement factor *α*; **c** linear gain coefficient *G*_g_.

**S4 Influence of continuous identical bits of digital signal on chaos synchronization**

There is a special case that a nonreturn-to-zero (NRZ) digital signal may be multiple identical modulation levels occur consecutively (such as 0000… or 1111…). In this case, the drive light is equivalent to a CW light without phase modulation, and thus the lasers tend to a stable state so that chaos synchronization is interrupted. Figure S5 depicts the time series of the modulation digital signal, the response signals, and the short-term cross-correlation with multiple identical modulation levels occur. It can be observed that when duration time of identical modulation levels is less than relaxation oscillation period (1/*f*_RO_) of the response laser, the correlation value between the response signals remains stable. Otherwise, the synchronization declines rapidly. The outputs of the response lasers cease to exhibit similarity, and only noise fluctuations can be observed in the time sequences. When the continuous-identical-level state disappears, the synchronization is recovered in a time scale of picoseconds. Increasing modulation rate can effectively shorten the bit duration time, and then reduce the synchronization interruption. Similarly, for the same baseband width, advanced modulation formats have more and shorter modulation levels than NRZ format, and thus can effectively reduce the probability of this synchronization interruption.


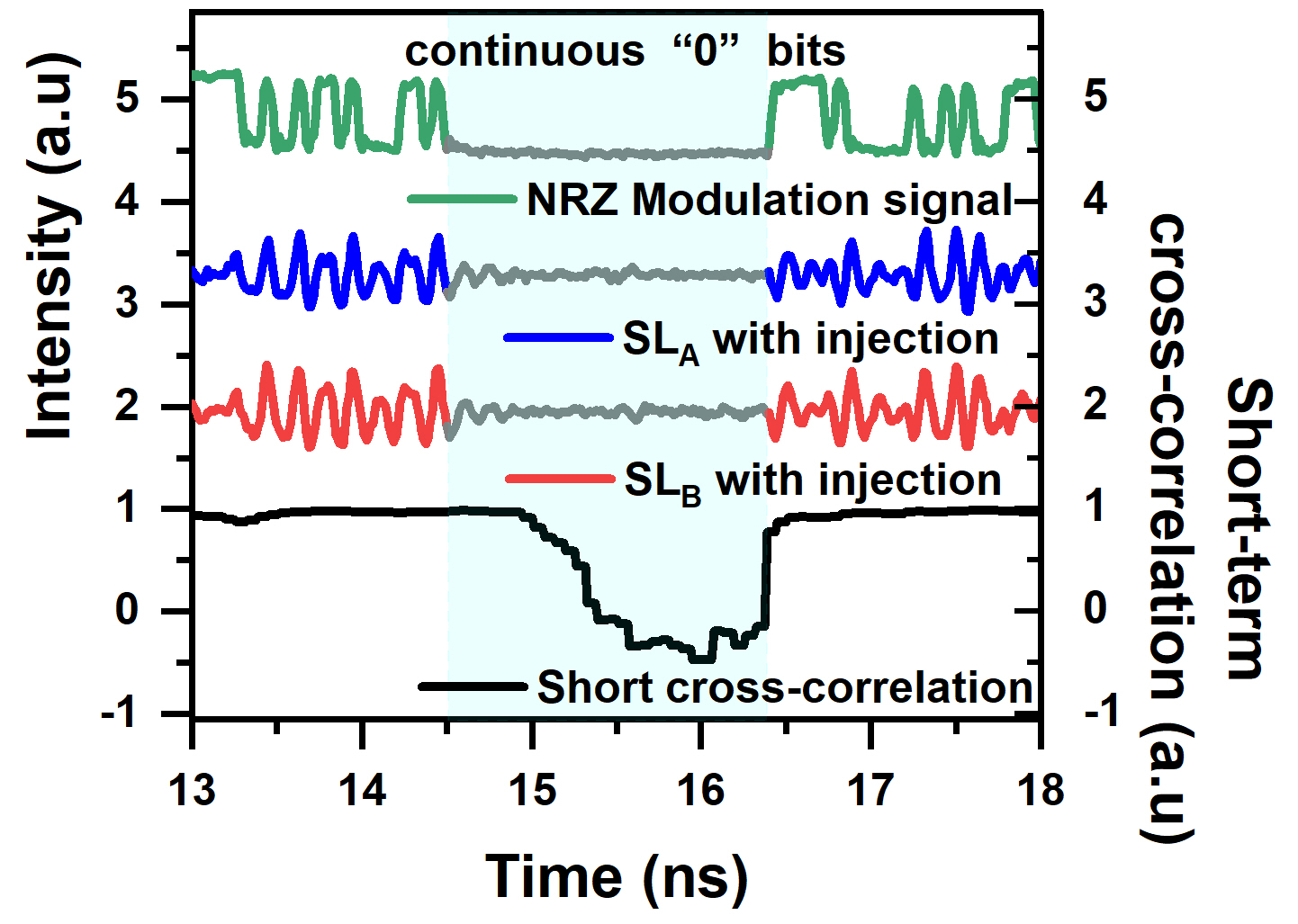


Fig. S5 Influence of continuous 0 bits of digital signal on synchronization. *κ*_j_=0.4, Δ*ν*_DR_=−2.5 GHz, *f*_m_=16 Gb/s, *φ*_m_=0.5π.

**References**

1. Lin, F. Y., Liu, J. Nonlinear dynamical characteristics of an optically injected semiconductor laser subject to optoelectronic feedback. *Optics Communications* **221** 173-180 (2012).
2. VPIphotonics. VPI component Maker photonic circuits user’ manual. https://www.vpiphotonics.com/Tools/ Photonic Circuits
3. Argyris, A., Grivas, E., Hamacher, M., Bogris, A. & Syvridis, D. Chaos-on-a-chip secures data transimission in optical fiber links. *Optics Express* **18** 5188-5198 (2010).
